# Exploratory Correlation Analysis of Respiratory Waveform, Instantaneous Respiratory Depth and Rate in Relation to Parasympathetic Indices During Spontaneous Breathing

**DOI:** 10.3390/bioengineering13030276

**Published:** 2026-02-27

**Authors:** Hironari Yagishita, Shota Tanabe, Hiroki Sato

**Affiliations:** Graduate School of Engineering and Science, Shibaura Institute of Technology, Saitama 337-8570, Japan; bn20261@shibaura-it.ac.jp (H.Y.); bn21201@shibaura-it.ac.jp (S.T.)

**Keywords:** respiratory depth, respiratory rate, parasympathetic, hilbert transform, spontaneous breathing

## Abstract

Respiration is closely related to the parasympathetic nervous system (PSNS). This relation occurs within a single respiratory cycle, with PSNS activity reduced during inspiration and increased during expiration. Over a longer timescale, deep and slow breathing has been reported to enhance PSNS activity, indicating that not only the timing (phase) but also the respiratory depth and rate may influence autonomic nervous system activity. However, under spontaneous breathing, it remains unclear which of the following best reflects PSNS activity: (i) raw respiratory waveform, (ii) respiratory depth, or (iii) respiratory rate. Respiratory depth was defined as instantaneous amplitude and respiratory rate as instantaneous frequency, both derived from the Hilbert transform. Respiratory waveforms and electrocardiograms were recorded at rest in 37 healthy adults. PSNS activity was quantified using heart rate variability indices reflecting parasympathetic modulation, including HF power, RMSSD, and CVI. Within-participant correlations between each respiratory measure and PSNS indices were obtained, and repeated-measures ANOVA with respiratory measure as a factor was used to compare correlation strengths. Results showed a significant main effect, with instantaneous amplitude consistently exhibiting significantly stronger correlations than the instantaneous frequency across all PSNS indices. These findings suggest that Hilbert-derived amplitude serves as a useful indicator of respiratory depth during spontaneous breathing and that depth is more strongly associated with PSNS activity.

## 1. Introduction

Respiration is closely linked to heart rate variability (HRV). One well-established manifestation of this link is respiratory sinus arrhythmia (RSA), characterized by an acceleration of heart rate during inspiration and deceleration during expiration [1]. This fluctuation occurs within a single respiratory cycle, reflecting reduced parasympathetic nervous system (PSNS) activity during inspiration and increased activity during expiration, thereby suggesting an association between respiratory phase and PSNS activity [2].

Beyond this short-term effect, deep and slow breathing has been reported to enhance PSNS activity over longer timescales [3]. Experimental work has further demonstrated that respiratory depth and rate exert independent influences on cardiac function [4,5]. Sroufe showed that respiratory rate affects only the stability of heart rate, with faster breathing reducing variability. In contrast, respiratory depth influences both the mean level and variability of heart rate, with deeper breathing tending to increase both [4]. These findings suggest that respiratory phase, depth, and rate may differentially modulate autonomic activity through distinct mechanisms.

Notably, many previous studies have relied on experimental manipulation of a single respiratory component, such as breathing frequency or tidal volume, in isolation [4,6,7]. Consequently, relatively few studies have examined the independent contributions of respiratory depth and respiratory rate when both fluctuate naturally within the same physiological signal. This limitation is important, as respiratory depth and frequency are regulated by partially distinct control mechanisms and may respond differently to cognitive demands, emotional states, or stressors [8,9].

To characterize respiratory dynamics during spontaneous breathing, respiratory depth and respiratory rate can be derived from the continuous respiratory signal using a Hilbert transform-based decomposition, following analytical approaches previously applied to electrophysiological signals [10,11,12]. The raw respiratory waveform contains mixed information related to respiratory phase, amplitude, and frequency, making it difficult to examine these components separately using the original signal alone. Conventional analyses of respiratory bellows signals often rely on peak or trough detection to estimate breath-by-breath respiratory depth and rate; however, such cycle-based approaches can be sensitive to noise, irregular breathing patterns, and ambiguity in cycle definition under spontaneous resting-state conditions [13].

Accordingly, the primary aim of the present study was to systematically compare the relationships between indices of parasympathetic nervous system activity and three representations of respiration—(i) the raw respiratory waveform, (ii) respiratory depth, and (iii) respiratory rate—derived from the same continuous respiratory signal during spontaneous resting-state breathing, using a Hilbert transform as a simple analytical approach. We hypothesized that, under these conditions, respiratory depth and respiratory rate would show differential associations with parasympathetic nervous system activity. Rather than proposing a novel physiological mechanism, the present study aims to provide a practical clarification of how different representations of respiration derived from the same continuous signal relate to parasympathetic indices during spontaneous breathing.

## 2. Materials and Methods

### 2.1. Participants

This study was approved by the Research Ethics Committee of Shibaura Institute of Technology (Approval No. 24-026), and written informed consent was obtained from all participants prior to the experiment. Eligibility criteria included the absence of any history of neurological or ophthalmological disorders, ensuring that participants could appropriately perceive the experimental instructions and complete both physiological measurements and questionnaires without difficulty.

A total of 37 university students participated in the study (20 males and 17 females; mean age = 21.1 ± 1.2 years). All acquired data were visually inspected, and data from four participants were excluded: two due to equipment malfunction that prevented data acquisition and two due to excessive noise in the physiological signals, which rendered further analysis infeasible. Consequently, data from 33 participants (17 males and 16 females) were included in the final analysis.

### 2.2. Data Acquisition and Analysis

Participants were seated in a chair and underwent physiological recordings during two consecutive resting conditions: 5 min with eyes closed followed by 5 min with eyes open. Physiological signals were recorded using a multi-channel biosignal acquisition system (MP6000, Miyuki Giken) at a sampling rate of 500 Hz. Only the eyes-open condition was included in the analyses, as this condition more closely reflects a typical waking state.

Electrocardiograms (ECGs) were recorded in lead II configuration with electrodes attached to the right wrist and left ankle. Respiration was recorded with a pneumatic belt sensor placed around the chest, capturing pressure changes associated with thoracic expansion and contraction. Respiratory bellows signals are commonly used to monitor breathing by measuring changes in chest or abdominal circumference and provide a noninvasive estimate of respiratory activity. Although chest belt signals do not directly measure airflow, previous validation studies have demonstrated that chest wall surface motion, when appropriately calibrated and measured under controlled resting conditions, reflects overall changes in tidal ventilatory volume in good agreement with reference airflow-based measurements [14,15]. However, belt-derived respiratory signals remain sensitive to factors such as body posture, belt placement, and breathing style. Accordingly, the chest belt signal was used to characterize time-varying respiratory activity rather than absolute ventilation.

Accordingly, respiratory features were derived using a Hilbert transform-based decomposition. The analytic signal obtained via the Hilbert transform was used to compute instantaneous amplitude and instantaneous frequency, which were operationally defined as indices of respiratory depth and respiratory rate, respectively, in accordance with prior work (Harrison, 2021) [13]. Using these Hilbert-derived measures, we examined whether respiratory depth and respiratory rate show differential associations with indices of parasympathetic nervous system activity during spontaneous resting-state breathing.

The preprocessing of respiratory signals followed the Hilbert transform-based method reported by Harrison et al. [13]. Specifically, low-frequency drifts (<0.01 Hz) and high-frequency noise (>2 Hz) were first removed, and the signal was subsequently low-pass filtered at 0.75 Hz to obtain a quasi-monocomponent waveform suitable for Hilbert decomposition, while preserving the dominant respiratory rhythm. The analytic signal was then derived as

(1) $$s_{a}\left(t\right)=s\left(t\right)+jH\left[s\left(t\right)\right]=s_{m}{(t)e}^{\left(j\phi \left(t\right)\right)},$$

which allows the original signal to be expressed as

(2) $$s\left(t\right)=s_{m}(t)\cos\left(\varphi \left(t\right)\right).$$

**Figure 1:**
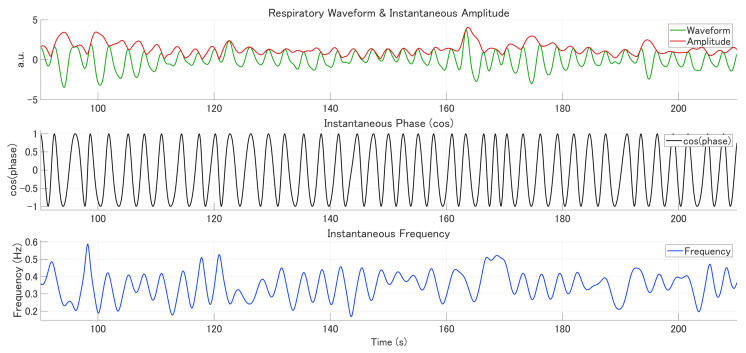
Respiratory Waveform with Corresponding Instantaneous Amplitude, Phase, and Frequency. This figure illustrates the signals from one representative participant. In the top panel, the respiratory waveform (green) and the instantaneous amplitude (red) are presented. The middle panel depicts the instantaneous phase, while the bottom panel represents the instantaneous frequency (blue).

The instantaneous amplitude *s_m_*(*t*) was defined as respiratory depth, while the instantaneous frequency,

(3) $$s_{f}\left(t\right)=\left(\frac{1}{2\pi }\right)\frac{d\varphi \left(t\right)}{dt}$$

was defined as respiratory rate. To correct for spurious negative frequencies, decreases in the instantaneous phase were linearly interpolated between local extrema, followed by additional low-pass filtering at 0.75 Hz. This phase-correction procedure was iterated ten times to ensure the monotonicity of the phase time course. Finally, Figure 1 illustrates representative examples of the respiratory signal after this preprocessing, showing the raw waveform together with the instantaneous amplitude, instantaneous phase, and instantaneous frequency derived from the Hilbert transform. Here, the raw respiratory waveform refers to the time-series signal recorded by the respiratory belt prior to Hilbert transform-based decomposition, reflecting thoracic or abdominal expansion and contraction over time.

To facilitate direct comparison across respiratory measures, the sign of the instantaneous frequency was inverted for interpretational consistency. From a physiological perspective, previous studies suggest that parasympathetic nervous system activity tends to increase during slow and deep breathing [4,16]. Accordingly, respiratory rate is generally expected to show a negative association with parasympathetic indices, whereas increases in respiratory depth are expected to show a positive association. Because the present study aimed to directly compare the relationships between these respiratory measures and PSNS indices, the sign of the instantaneous frequency was inverted after frequency estimation.

R–R intervals were extracted from the ECG using the Pan–Tompkins algorithm [17,18]. R–R intervals outside the physiological range (250–1500 ms) were treated as missing values and interpolated using cubic splines [19,20]. PSNS indices were then calculated from the R–R interval time series.

Frequency-domain analysis was performed using the Lomb–Scargle periodogram to estimate spectral power in the high-frequency (HF; 0.15–0.40 Hz) band [21]. HF power, reflecting vagal modulation of the heart, was log-transformed to correct for non-normality [22,23]. In the time domain, the root mean square of successive differences (RMSSD) was computed as an index of cardiac vagal activity [24]. In addition, the Toichi cardiac vagal index (CVI), defined as log10(SD1 × SD2) derived from the Poincaré plot, was calculated as another measure of PSNS modulation [25].

Continuous HRV measures were calculated using a 5 s sliding window with PSNS indices computed beat by beat. The interpolated R–R interval series was resampled at 0.1 s intervals using piecewise cubic Hermite interpolating polynomial. The choice of a 5 s window was based on a balance between temporal resolution and reliability, yielding a strong correlation with HRV measures computed over the entire recording (HF: r = 0.90, RMSSD: r = 0.99, CVI: r = 0.92). This approach is supported by previous studies showing that, although classification performance generally improves with longer windows, ultra-short windows on the order of 5 s can still retain sufficient discriminative information for detecting cognitive load states [26].

Within each participant, Spearman’s correlation coefficients were calculated between each respiratory measure (respiratory waveform, instantaneous amplitude, instantaneous frequency) and PSNS indices (HF, RMSSD, CVI).

For each PSNS index (HF, RMSSD, CVI), repeated-measures analysis of variance (ANOVA) was conducted on Fisher z-transformed correlation coefficients to compare the strength of associations with different respiratory measures. Post hoc multiple comparisons were performed using the Bonferroni correction. These analyses allowed us to statistically evaluate whether the type of respiratory measure significantly influenced the strength of its association with PSNS indices.

## 3. Results

### 3.1. Respiratory Features and Their Associations with Parasympathetic Nervous System Indices

**Figure 2:**
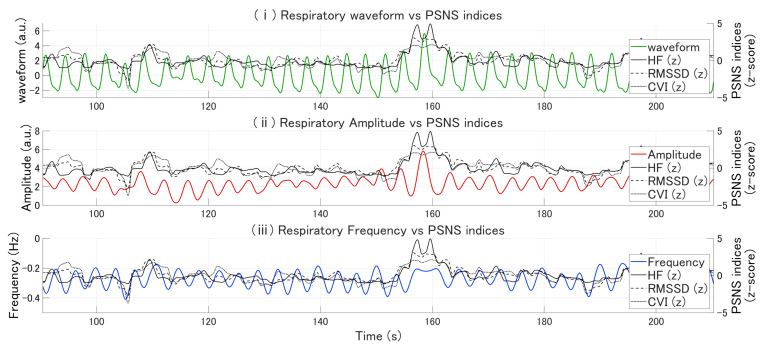
Respiratory features and their temporal associations with parasympathetic nervous system (PSNS) indices. (Panel (**i**)) Respiratory waveform (green) plotted alongside PSNS indices: high-frequency power of heart rate variability (HF; solid line), root mean square of successive differences in R–R intervals (RMSSD; dashed line), and cardiac vagal index (CVI; dotted line). (Panel (**ii**)) Respiratory instantaneous amplitude (red) plotted against the same PSNS indices. (Panel (**iii**)) Respiratory instantaneous frequency (blue) plotted against the same PSNS indices.

Figure 2 illustrates the temporal relationships between respiratory measures and PSNS indices for representative participants. Notably, deep breathing events were accompanied by increases in HF, RMSSD, and CVI, suggesting a close temporal association between PSNS activity and respiratory depth.

### 3.2. Correlation Coefficients Between Respiratory Measures and PSNS Indices

**Figure 3:**
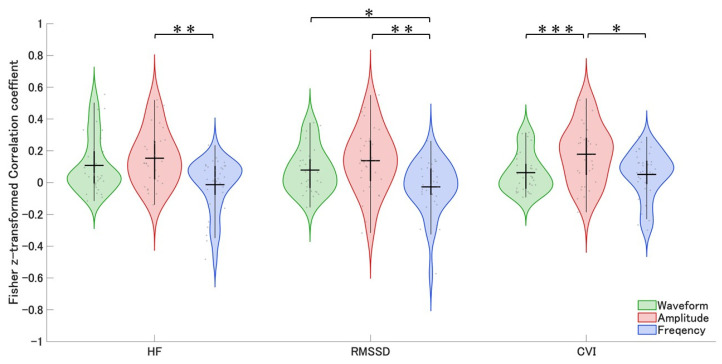
Correlation coefficients between respiratory measures and PSNS indices. Violin plots show the distributions of Fisher z-transformed correlation coefficients between three respiratory measures—the respiratory waveform (green), respiratory instantaneous amplitude (red), and respiratory instantaneous frequency (blue)—and three PSNS indices (HF power, RMSSD, and CVI). Individual participants are shown as gray dots, and black bars indicate mean ± standard error. Asterisks indicate significant differences between respiratory measures based on repeated-measures ANOVA with Bonferroni correction (* *p* < 0.05, ** *p* < 0.01, *** *p* < 0.001).

Figure 3 summarizes the group-level comparisons across respiratory measures. A repeated-measures ANOVA revealed a significant main effect of respiratory measure on HF (F(2, 64) = 6.92, *p* = 0.0019, partial η^2^ = 0.18). Bonferroni-corrected pairwise comparisons showed that instantaneous amplitude showed significantly higher correlation coefficients than instantaneous frequency (mean difference = 0.166, SE = 0.051, *p* = 0.0077). No significant differences were observed between instantaneous amplitude and the respiratory waveform, or between the respiratory waveform and instantaneous frequency.

For RMSSD, the main effect of respiratory measure was also significant (F(2, 64) = 8.31, *p* = 0.00062, partial η^2^ = 0.21). Pairwise comparisons revealed that instantaneous amplitude was significantly greater than instantaneous frequency (mean difference = 0.165, SE = 0.049, *p* = 0.0060). In addition, the respiratory waveform was significantly greater than instantaneous frequency (mean difference = 0.106, SE = 0.042, *p* = 0.0499). No significant difference was found between instantaneous amplitude and the respiratory waveform.

Similarly, for CVI, a significant main effect of respiratory measure was observed (F(2, 64) = 7.29, *p* = 0.0014, partial η^2^ = 0.19). Bonferroni-corrected pairwise comparisons indicated that instantaneous amplitude was significantly greater than instantaneous frequency (mean difference = 0.127, SE = 0.046, *p* = 0.0268) and also significantly greater than the respiratory waveform (mean difference = 0.116, SE = 0.026, *p* = 0.00026). No significant difference was observed between the respiratory waveform and instantaneous frequency.

Across all parasympathetic nervous system indices (HF, RMSSD, and CVI), instantaneous amplitude consistently showed significantly higher values than instantaneous frequency, with medium-to-large effect sizes. Additional analyses examining sex differences and eye condition (eyes open vs. eyes closed) revealed no significant effects on the correlation coefficients (Appendix A.1 and Appendix A.2).These results indicate that respiratory depth, indexed by instantaneous amplitude, shows stronger associations with short-term parasympathetic cardiac modulation than respiratory rate.

## 4. Discussion

This study showed that, under spontaneous breathing, respiratory depth indexed by the instantaneous amplitude derived from the Hilbert transform was more strongly associated with temporal fluctuations in parasympathetic nervous system activity. These patterns further suggest that, even during spontaneous breathing, transient deep inhalations were associated with short-lived increases in parasympathetic indices, without corresponding changes in respiratory rate. In particular, the group-level differences observed across all PSNS indices suggest that increased lung expansion and the associated changes in intrathoracic pressure may be more closely associated with vagal reflexes supporting respiration-related modulation of heart rate [27]. Moreover, because the instantaneous amplitude obtained from the Hilbert transform represents the envelope of the respiratory waveform, it allows the extraction of intensity-related components without being strongly affected by phase reversals. Consequently, compared with the raw respiratory waveform, which includes phase-dependent polarity changes, this measure may more faithfully reflect vagally mediated amplitude fluctuations.

In contrast, the instantaneous frequency (respiratory rate) showed weaker associations with PSNS indices. One possible explanation is that resting spontaneous respiratory rate typically fluctuates within a relatively narrow range around 0.2–0.3 Hz, thereby reducing statistical sensitivity [28]. Furthermore, when the respiratory rate lies outside the HF band (0.12–0.40 Hz), HF power no longer accurately reflects vagal regulation, rendering the estimation of respiratory sinus arrhythmia unstable [29]. Higher respiratory rates are also often accompanied by stress-related responses and increased sympathetic activity, which may exert opposing effects on PSNS indices [30,31]. Thus, multiple confounding factors may obscure the relationship between respiratory rate and RSA-related measures.

Several limitations of this study should be acknowledged.

First, because measurements were conducted under spontaneous breathing conditions, it is difficult to strictly dissociate whether changes in respiratory patterns preceded or drove fluctuations in parasympathetic activity. Although resting spontaneous breathing closely approximates natural breathing, both respiratory rate and depth may vary simultaneously, limiting the ability to disentangle their independent effects.

Second, although PSNS indices were derived from HRV, HRV primarily reflects cardiac vagal modulation and therefore requires cautious interpretation. The use of HRV as a surrogate marker of cardiac vagal tone remains a subject of ongoing debate and involves several methodological considerations [32]. In addition, the present sample was limited to healthy young adults, and all measurements were obtained under a single resting condition. Accordingly, the generalizability of the present findings to older adults or clinical populations cannot be established.

Moreover, the menstrual cycle phase of female participants was not assessed in the present study. Given that fluctuations in ovarian hormones have been reported to influence autonomic nervous system activity, this factor should be considered when interpreting the results [33,34]. In addition, RSA amplitude is known to decline with age, with the most pronounced decrease occurring between the third and fourth decades of life [35]. Therefore, the relationship between respiration and RSA may differ in older populations.

Third, several key physiological and state-related variables, including tidal volume and end-tidal carbon dioxide, were not directly measured, precluding evaluation of the contribution of ventilatory chemoreflexes. Previous studies have shown that hypercapnia increases heart rate, tidal volume, and RSA amplitude, thereby altering the relationship between vagal tone and RSA [36].

Based on these considerations, future research should address the following issues. (1) To validate the amplitude-based index used in this study, direct comparisons between Hilbert-derived amplitude estimates and ventilatory measures such as tidal volume or airflow are required to determine whether this index reliably reflects time-varying respiratory activity during spontaneous breathing. (2) Experimental paradigms should be designed to independently manipulate respiratory depth and respiratory rate. Because simultaneous changes in depth and rate can produce complex RSA responses, fixing one parameter while manipulating the other will help clarify their independent contributions to RSA and PSNS activity. (3) Future studies should include a wider range of age groups and clinical populations to evaluate the generalizability of the observed respiration–RSA relationships.

## 5. Conclusions

This study showed that, under spontaneous breathing, respiratory depth indexed by the instantaneous amplitude derived from the Hilbert transform was more strongly associated with fluctuations in parasympathetic nervous system activity than respiratory rate. In particular, instantaneous amplitude consistently yielded stronger associations than instantaneous respiratory rate across all PSNS indices, including HF, RMSSD, and CVI, suggesting that respiratory depth provides a more sensitive representation of vagally mediated cardiac modulation during rest.

In contrast, instantaneous respiratory rate showed less robust associations with PSNS indices, likely reflecting the relatively narrow range of spontaneous breathing frequencies at rest and their susceptibility to confounding factors such as arousal-related sympathetic activation.

Together, these findings provide insights into the interaction between respiration and heart rate variability and highlight the methodological advantage of using Hilbert-derived respiratory amplitude to characterize RSA and short-term parasympathetic activity. Future research should incorporate direct measurements of tidal volume and expired CO_2_ and employ experimental paradigms that independently manipulate respiratory depth and respiratory rate to clarify their distinct contributions to RSA and PSNS regulation.

## Figures and Tables

**Figure A1:**
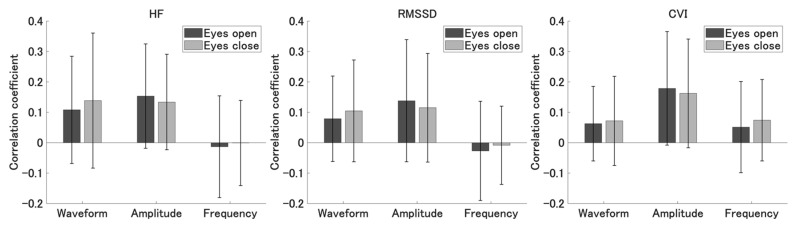
Comparison between eyes-open and eyes-closed conditions. Group-level correlation coefficients (mean ± SD) are shown for the eyes-open and eyes-closed states across respiratory waveform, amplitude, and frequency representations. Results are presented separately for parasympathetic indices (HF, RMSSD, and CVI).

**Figure A2:**
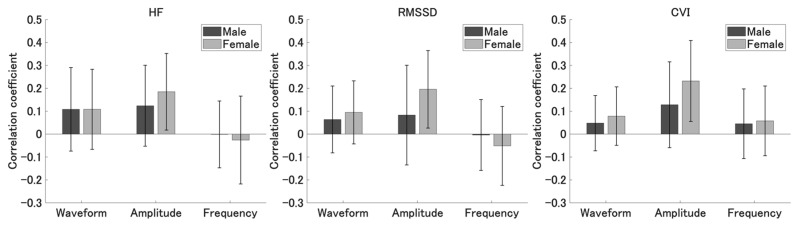
Comparison between male and female participants. Group-level correlation coefficients (mean ± SD) are shown for males and females across respiratory waveform, amplitude, and frequency representations. Results are presented separately for parasympathetic indices (HF, RMSSD, and CVI).

## Data Availability

The data supporting the findings of this study are available from the corresponding author upon request.

## Abbreviations

The following abbreviations are used in this manuscript:

HRV	Heart Rate Variability
RSA	Respiratory Sinus Arrhythmia
PSNS	Parasympathetic Nervous System
EEG	Electroencephalogram
ECG	Electrocardiogram
RMSSD	Root Mean Square of Successive Differences
HF	High-frequency
CVI	Cardiac Vagal Index
ANOVA	Analysis of Variance

## Appendix A

### Appendix A.1. Comparison Between Eyes-Open and Eyes-Closed Conditions

An additional analysis was conducted to examine potential differences between eyes-open and eyes-closed conditions.

Correlation measures were computed separately for the eyes-open and eyes-closed states using the same preprocessing and analysis pipeline as described in the main Methods. Respiratory waveform, amplitude, and frequency representations were evaluated for parasympathetic indices, including HF, RMSSD, and CVI.

Statistical comparisons between eyes-open and eyes-closed conditions were performed using paired-sample *t*-tests, as both conditions were obtained from the same participants. All statistical tests were two-tailed, and the same criteria for statistical significance as in the main analyses were applied.

No statistically significant differences were observed between the eyes-open and eyes-closed conditions for any of the examined measures. This absence of significant effects was consistent across all representations (respiratory waveform, amplitude, and frequency) and across all parasympathetic indices (HF, RMSSD, and CVI), as shown in Figure A1.

These results indicate that the correlation patterns reported in the main analyses were not systematically affected by the visual state (eyes open or eyes closed).

### Appendix A.2. Comparison Between Male and Female Participants

An additional analysis was conducted to examine potential differences between male and female participants.

Correlation measures were computed using the same preprocessing and analysis pipeline as described in the main Methods. Respiratory waveform, amplitude, and frequency representations were evaluated for parasympathetic indices, including HF, RMSSD, and CVI.

Statistical comparisons between male and female participants were performed using independent-sample *t*-tests, as the two groups consisted of different participants. All statistical tests were two-tailed, and the same criteria for statistical significance as in the main analyses were applied.

No statistically significant differences were observed between male and female participants for any of the examined measures. This absence of significant effects was consistent across all representations (respiratory waveform, amplitude, and frequency) and across all parasympathetic indices (HF, RMSSD, and CVI), as shown in Figure A2.

These results indicate that the correlation patterns reported in the main analyses were not systematically affected by participant sex.
